# Untargeted metabonomics and TLR4/ NF-κB signaling pathway analysis reveals potential mechanism of action of *Dendrobium huoshanense* polysaccharide in nonalcoholic fatty liver disease

**DOI:** 10.3389/fphar.2024.1374158

**Published:** 2024-06-03

**Authors:** Guang-hui Deng, Chen-chen Zhao, Xiao Cai, Xiao-qian Zhang, Meng-zhen Ma, Jia-hui Lv, Wen-li Jiang, Dai-yin Peng, Yan-yan Wang, Li-hua Xing, Nian-jun Yu

**Affiliations:** ^1^ School of Pharmacy, Anhui University of Chinese Medicine, Hefei, China; ^2^ Anhui Academy of Traditional Chinese Medicine and Institute of Conservation and Development of Traditional Chinese Medicine Resources, Hefei, China; ^3^ MOE-Anhui Joint Collaborative Innovation Center for Anhui Genuine Chinese Medicinal Materials, Hefei, China

**Keywords:** Dendrobium huoshanense, nonalcoholic fatty liver disease, NF-κB, polysaccharide, metabolomics

## Abstract

Nonalcoholic fatty liver disease (NAFLD) is marked by hepatic steatosis accompanied by an inflammatory response. At present, there are no approved therapeutic agents for NAFLD. *Dendrobium Huoshanense* polysaccharide (DHP), an active ingredient extracted from the stems of *Dendrobium Huoshanense*, and exerts a protective effect against liver injury. However, the therapeutic effects and mechanisms of action DHP against NAFLD remain unclear. DHP was extracted, characterized, and administered to mice in which NAFLD had been induced with a high-fat and high-fructose drinking (HFHF) diet. Our results showed that DHP used in this research exhibits the characteristic polysaccharide peak with a molecular weight of 179.935 kDa and is composed primarily of Man and Glc in a molar ratio of 68.97:31.03. DHP treatment greatly ameliorated NAFLD by significantly reducing lipid accumulation and the levels of liver function markers in HFHF-induced NAFLD mice, as evidenced by decreased serum levels of aspartate aminotransferase (AST), alanine aminotransferase (ALT), total cholesterol (TC) and total triglyceride (TG). Furthermore, DHP administration reduced hepatic steatosis, as shown by H&E and Oil red O staining. DHP also inhibited the Toll-like receptor 4 (TLR4)/nuclear factor-kappa B (NF-κB) signaling pathway expression, thereby reducing levels of hepatic proinflammatory cytokines. Besides, untargeted metabolomics further indicated that 49 metabolites were affected by DHP. These metabolites are strongly associated the metabolism of glycine, serine, threonine, nicotinate and nicotinamide, and arachidonic acid. In conclusion, DHP has a therapeutic effect against NAFLD, whose underlying mechanism may involve the modulation of TLR4/NF-κB, reduction of inflammation, and regulation of the metabolism of glycine, serine, threonine, nicotinate and nicotinamide metabolism, and arachidonic acid metabolism.

## 1 Introduction

Nonalcoholic fatty liver disease (NAFLD), a chronic hepatic manifestation of the metabolic syndrome, is a critical global health challenge (Estes et al., 2018). NAFLD is characterized by the deposition of fat ectopically in the liver, without the involvement of excessive drinking or other factors that may cause liver damage (Wesolowski et al., 2017). NAFLD is prevalent in about 25% in the general population and up to 75%–100% in obese individuals, with an increasing trend (Younossi et al., 2016; Huang et al., 2021). To date, there are no approved effective pharmacological therapies for NAFLD are available and controlling the associated complications has limited beneficial effects. Therefore, it is critical to identify effective experimental drugs to treat NAFLD.

*Dendrobium huoshanense* C. Z. Tang et S. J. Cheng, an edible and medicinal plant in the family Orchidaceae, possesses excellent medicinal properties that benefit nourish Yin, and eliminate heat (Hsieh et al., 2008; Yu et al., 2022). *Dendrobium* *huoshanense* polysaccharide (DHP) is the major component, with an array of biological properties, including anti-inflammatory, anti-oxidation, and hepatoprotective activities (Pan et al., 2012; Li et al., 2015; Fu et al., 2023). Researchers have indicated that DHP protects against liver injury by inhibiting inflammation, as well as attenuating hepatic injury and fibrosis (Zha et al., 2006; Pan et al., 2012; Wang et al., 2014; Wang et al., 2015). However, whether DHP protects against NAFLD, and the potential mechanisms of action involved, have not been clarified.

Metabolomics is the study of the dynamic endogenous metabolites in organs, organ systems or organisms (Johnson et al., 2016). Numerous researchers have applied untargeted metabolomics to explore the incidence and progression of NAFLD (Masoodi et al., 2021; Cheng et al., 2022). Although liver metabolites and metabolic pathways are known to be significantly altered in mice with NAFLD, few studies have applied a liver metabolomics approach to investigate how DHP can mitigate the effects of this disease.

Toll-like receptor 4 (TLR4) and nuclear factor-κB (NF-κB) have been implicated in the regulation of inflammatory cytokines such as interleukin-6 (IL-6), interleukin-1β (IL-1β) and tumor necrosis factor-α (TNF-α) (Zhao et al., 2017). Moreover, NAFLD is related to chronic low-grade systemic inflammation, which causes hepatic steatosis to nonalcoholic steatohepatitis (NASH) (Tilg et al., 2021). Several studies have indicated that activation of TLR4/NF-κB activation is a primary factor in the induction of hepatic inflammation due to liver injury in NAFLD (Dela Peña et al., 2005; Mu et al., 2021). Thus, inhibition of the TLR4/NF-κB signaling pathway is a potential therapeutic approach for NAFLD. The current study aimed to investigate the effects of DHP on NAFLD and to explore the underlying mechanisms via untargeted metabolomics and analysis of the TLR4/NF-κB signaling pathway.

## 2 Materials and methods

### 2.1 Preparation of DHP

Dendrobium huoshanense plant stems were collected from the Huoshan County, Anhui Province of China. Referring to the subject group’s pre-extraction method (Ye et al., 2022), the powder was degreased with 95% ethanol at 25°C for 24 h, centrifuged, and retained. Then, the Sevage method was used to deproteinize the precipitates after extraction with distilled water. Impurities with molecular weights less than 3,500 Da were removed by dialysis. Eventually, DHP was obtained by freeze-drying. The structure of DHP was resolved using SEM, FT-IR, HPGPC, and ion chromatography (IC). Specific experimental steps are described in Supplementary Material S1.

### 2.2 NAFLD mice model

C57BL/6J mice aged five to 8 weeks were maintained in cages under standard laboratory conditions (20°C–25°C temperature, 40%–70% relative humidity). The mice were fed a high-fat diet (fat, 60%; carbohydrates, 20%; protein, 20%; Jiangsu Xietong Pharmaceutical Bio-engineering Co., Ltd) and high-fructose drinking solution (10% D-fructose, Shanghai Macklin Biochemical Co., Ltd) diet for 12 weeks to establish the NAFLD mouse model.

**FIGURE 1:**
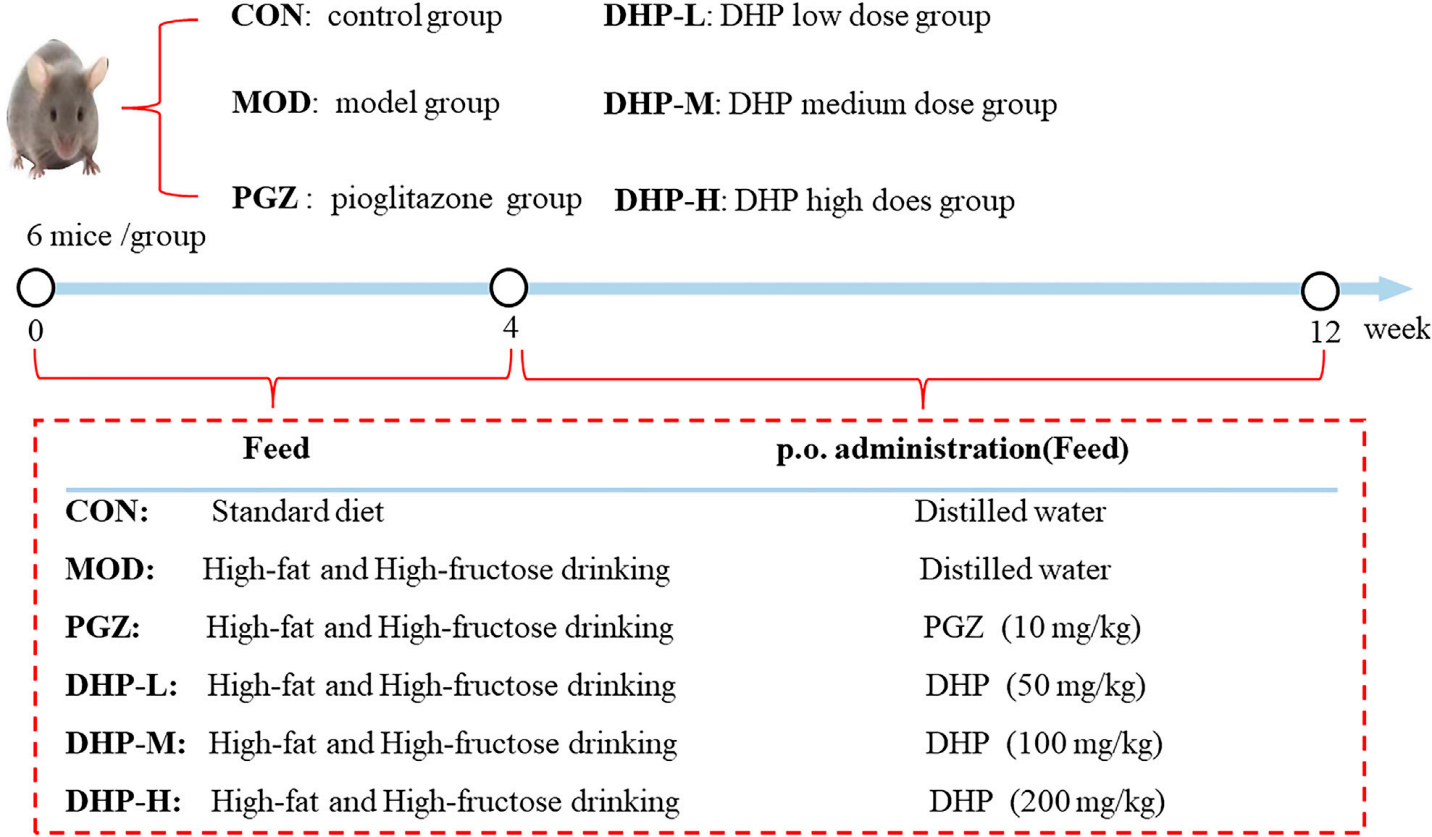
Animal experimental protocol for this research.

As illustrated in Figure 1, the mice were allocated into six distinct groups (n = 6 mice per group) in a random manner: the control group (CON), the model group (MOD), the pioglitazone group (PGZ, 10 mg/kg), the DHP-low dose group (DHP-L, 50 mg/kg), the DHP-medium dose group (DHP-M, 100 mg/kg), and the DHP-high dose group (DHP-H, 200 mg/kg). All mice, except the CON, were fed High-Fat and High-Fructose drinking (HFHF) and administered the various drugs in the period from weeks 5–12.

At the 4 weeks feeding time point, mice in DHP-L, DHP-M and DHP-H groups were injected with 50 mg/kg, 100 mg/kg and 200 mg/kg of DHP by gavage once a day, respectively, mice in the PGZ group were given with 10 mg/kg PGZ per day and MOD mice were given the same dose of the saline for 8 weeks. CON mice were fed standard chow (fat, 11.1%; carbohydrates, 67.4%; protein, 21.5%; Jiangsu Xietong Pharmaceutical Bio-engineering Co., Ltd 3) and normal water and were given with saline after the fourth week according to their body weight. Before euthanasia, the mice were fasted overnight. Euthanasia was performed by intraperitoneal injection of 3% pentobarbital. Blood was then collected from the abdominal aorta, centrifuged at 1,500 rpm, 4°C, for 15 min, and the supernatant was collected. The mouse livers were collected in lyophilized tubes and stored at −80°C. This study was approved by the Ethics Committee of the Anhui University of Chinese Medicine.

### 2.3 Body weight and liver index calculation

The body weight of the mice were recorded on a weekly basis. The liver index was calculated using the following formula: Liver index = liver weight (mg)/body weight g).

### 2.4 Histological analysis

Liver tissues were prepared in 4 μm tissue sections by conventional paraffin embedding, stained according with hematoxylin and eosin (H&E) and Oil Red O, viewed under a microscope, and photographed.

### 2.5 Analysis of biochemical parameters in serum

The levels of TC, TG, ALT, AST, low-density lipoprotein cholesterol (LDL-C), and high-density lipoprotein cholesterol (HDL-C) in the serum were calculated using a fully automated biochemical analyzer (Chemray-240).

### 2.6 Enzyme-linked immunosorbent assay

Liver tissues were homogenized in RIPA buffer. Then, the sample was vortexed and centrifuged at 2000 rpm for 5 min at 4°C. The supernatant was collected and stored at 80°C before performing ELISA. TC, TG, TNF-α, IL-1β, and IL-6 were detected in mouse livers using the corresponding kits (ColorfulGene Biological Technology Co.,Ltd (Wuhan, China) according to the manufacturer’s instructions.

### 2.7 Untargeted metabolome analysis

Liver tissue (30 mg) was placed in 400 μL of methanol-water (V: V = 7:3) solution, ground, and centrifuged. Then, the supernatant was collected for LC-MS analysis. The separation was carried out on the Waters Acquity UPLC HSS T3 C18 column (2.1 *100 mm, 1.8 µm) at 40°C with mobile phases A and B consisting of water and acetonitrile with 0.1% formic acid, respectively. The gradient elution procedure was as follows: within 12 min, mobile phase A was reduced from 95% to 10%, held for 1 min at 10%, and then returned to 95% for 3 min. For mass spectrometry measurements, an Agilent Q-TOF-6545 mass spectrometer was used with an interface voltage of 2.5 kV for positive ions and −1.5 kV for negative ions.

For analysis of metabolites, m/z values, formulas, and MS/MS fragmentations were compared with online databases, including Kyoto Encyclopedia of Genes and Genomes (KEGG) database (http://www.kegg.jp/), the Human Metabolome Database (HMDB) (http://www.hmdb.ca), and PubChem (https://puccbi.nlm.nih.gov/), among others. MetaboAnalyst 5.0 (https://www.metaboanalyst.ca/MetaboAnalyst/) was applied to conduct an orthogonal partial least squares discriminant analysis (OPLS-DA), partial least squares discriminant analysis (PLS-DA), and principal component analysis (PCA). Using MetaboAnalyst 5.0, we screened differential metabolites by the variable importance in projection (VIP) > 1 and the *p* < 0.05, followed by metabolic pathway analysis.

### 2.8 RT-PCR analysis

Total RNA was extracted from liver tissue and reverse-transcribed into cDNA with the SPARK script II RT Plus Kit (SparkJade Co., Ltd. Shandong, China), using Sangon Biotech synthesized primers (Sangon Biotech Co., Ltd. Shanghai, China) (Table 1). SYBR Green in a Light Cycler@480 System (Roche, Switzerland) was used to perform qRT-PCR analysis.

**TABLE 1:** Real-time PCR primer sequences.

Gene	Forward primer	Reverse primer
β-actin	GTG​CTA​TGT​TGC​TCT​AGA​CTT​CG	ATG​CCA​CAG​GAT​TCC​ATA​CC
TNF-α	ACC​CTC​ACA​CTC​ACA​AAC​CAC	ACA​AGG​TA.CAA​CCC​ATC​GGC
IL-1β	GCC​ACC​TTT​TGA​CAG​TGA​TGA​G	TGA​TGT​GCT​GCT​GCG​AGA​TT
IL-6	AGA​CAA​AGC​CAG​AGT​CCT​CCA​G	GTGACTCCAGCZTTATCTCTTCCT

### 2.9 Immunofluorescence staining

Liver tissues from the mice ×were fixed in formalin solution, cut into paraffin sections, deparaffinized, and hydrated for antigenic repair. F4/80, CD68, and p-NF-κB primary antibodies (1:200) were incubated overnight at 4°C before being washed with PBS (5 min × 3 times) (Cell Signaling Technology, USA). Following incubation with the primary antibodies, a secondary antibody (1:300) was added, followed by incubation for 1 h at room temperature. Sections were then washed with PBS (5 min × 3 times). In the final step of the process, after the surface of the tablet had been treated with a quenching agent that inhibits fluorescence, the tablet was sealed. Staining was visualized and photographed using a fluorescence microscope (Leica, Germany).

### 2.10 Western blot analysis

Fresh liver tissues were acquired and ground, then, RIPA buffer was added for further grinding. Next, bicinchoninic acid assay was performed to determine the protein concentration in the supernatant after centrifugation of the total protein extract. SDS-PAGE gels were prepared. After sampling, electrophoresis and membrane transfer, then, was performed; primary antibodies (TLR4 (ZENBIO, China), MyD88 (Proteintech, China), NF-Κb (Proteintech, China), p-NF-κB, and β-actin (ZENBIO, China) were used at the appropriate dilution, followed by incubation overnight at 4°C in the refrigerator. A secondary antibody solution was added to the strips, and they were incubated at room temperature for 2 h. Enhanced chemiluminescence (ECL) reagents were used to detect antigen-antibody complexes, visualized using an ECL chemiluminescence imaging system (G&E Healthcare, USA). Grayscale analysis was conducted using Image Lab.

### 2.11 Statistical analysis

All experimental results were expressed as the means ± standard deviation (mean ± SD). GraphPad Prism 9.0 was used to analyze the data. The difference between multiple groups was compared using a one-way ANOVA (One-way ANOVA), with *p* < 0.05 indicating statistical significance.

## 3 Results

### 3.1 Structural analysis of DHP

**FIGURE 2:**
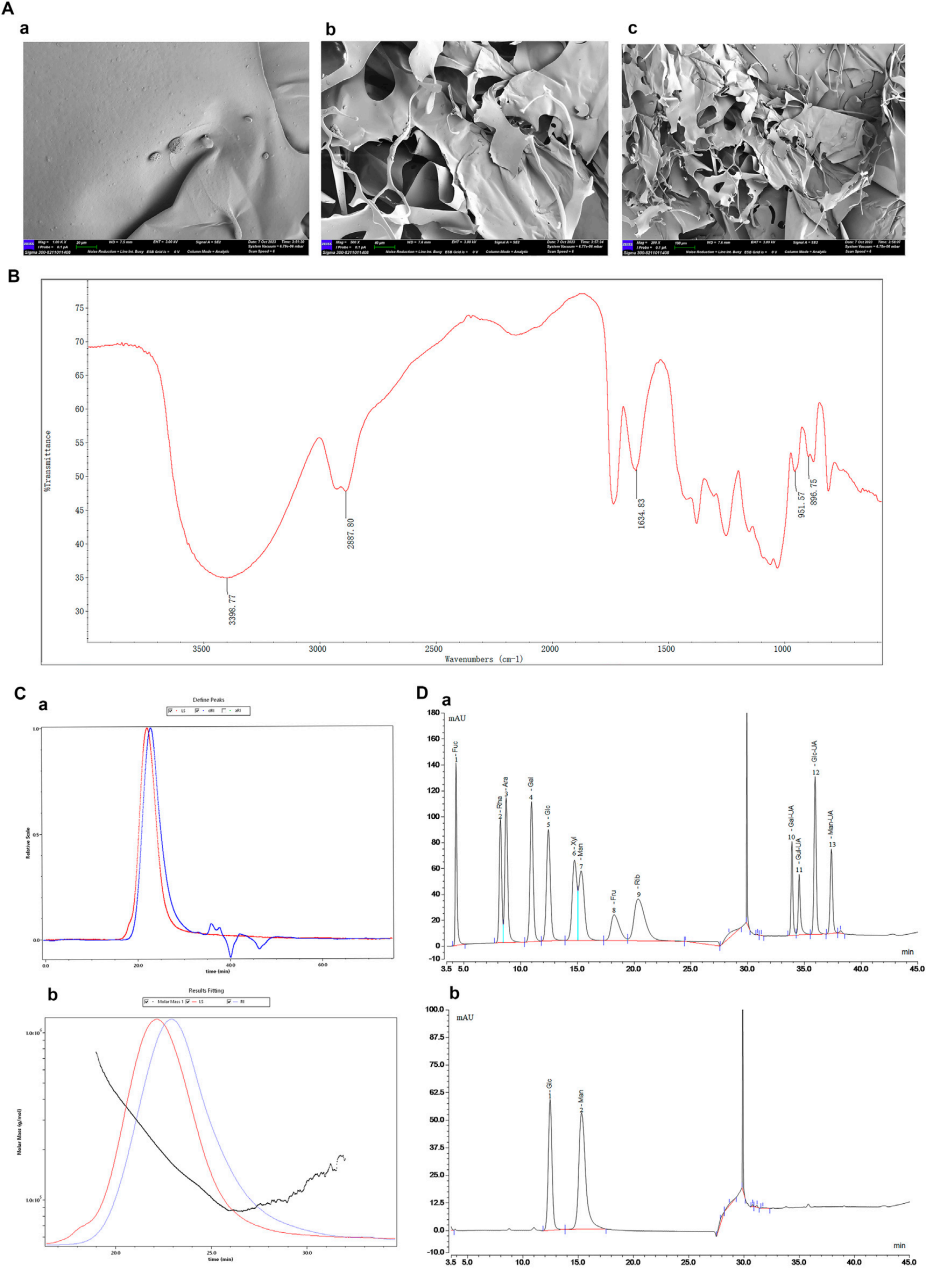
Structural analysis of DHP. **(A)** SEM (a:1.00 kx, **(B) **500 x, **(C)**200x). **(B)** FT-IR. **(C)** Molecular weight (a: HPGPC analysis of DHP, **(B)** Absolute molecular weight analysis). **(D)** Monosaccharide composition (a: Ion chromatogram of monosaccharide, **(B)** Ion chromatogram of the DHP). 1. Fucose (Fuc). 2. Rhamnose (Rha). 3. Arabinose (Ara). 4. Galactose (Gal). 5. Glucose (Glc). 6. Xylose (Xyl). 7. Mannose (Man). 8. Fructose (Fru). 9. Ribose (Rib). 10. Galacturonic acid (Gal-UA). 11. Glucuronic acid (Glc-UA). 12. Glucuronic Acid (Glc-UA).13 Mannuronic Acid (Man-UA).

The molecular morphology of DHP was examined using a scanning electron microscope and observed at image magnifications of 1.00 k×, 500.00 ×, and 200.00 ×. Microstructural analysis of DHP revealed an irregular lamellar structure with an uneven surface (Figure 2A). As shown in Figure 2B, the DHP exhibited strong absorption peaks at 3,398 cm^-1^ and 2,887 cm^-1^, which were caused by stretching vibration of the O–H and the C–H bond, respectively, and were polysaccharides-specific peaks. The characteristic absorption peaks at 951 cm^-1^ and 896 cm^-1^ indicates that the DHP has both the typical α-configuration and β-configuration. As shown in Figure 2A-C, the weight and polydispersity index (Mw/Mn) and number-average molecular weight (Mw and Mn) of DHP were determined using SEC-MALLS-RI, as 179.935 kDa, 1.224 and 146.96 kDa, 1.224, respectively. The purity of DHP was 89.55%. Being a heteropolysaccharide composed of Man and Glc, DHP was found to consists of 68.97% and 31.03% mannose and glycoside, respectively.

### 3.2 *Dendrobium huoshanense* polysaccharide ameliorates NAFLD

**FIGURE 3:**
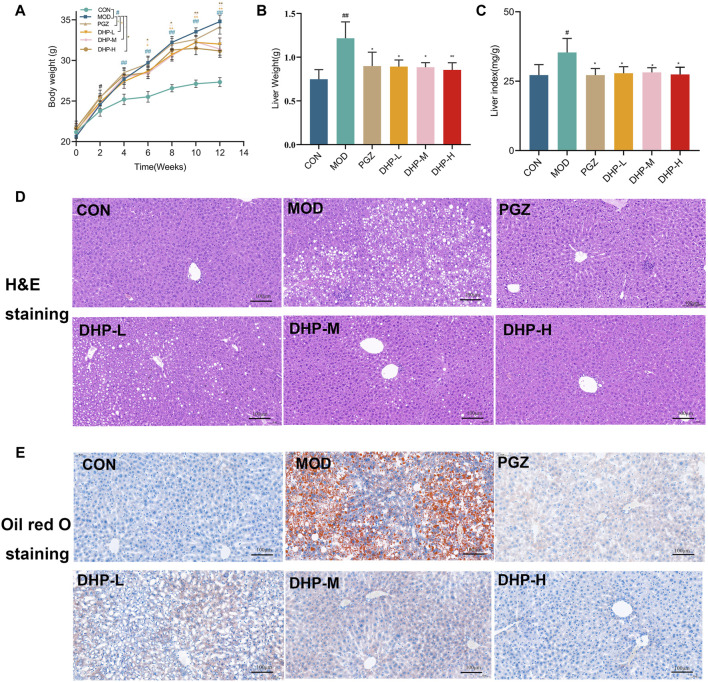
DHP alleviates hepatic steatosis and injury in HFHF-induced NAFLD mice. **(A)** Body weight. **(B)** Liver weight. **(C)** Liver index. **(D)** Liver histopathology was assessed by H&E staining (200×, 100 μm). **(E)** Oil red O staining of liver tissues (200×, 100 μm). Data are presented as mean ± standard deviation (mean ± SD, n = 6). ^#^ *p* < 0.05, ^##^ *p* < 0.01 vs. the control group. *p < 0.05, **p < 0.01, vs. the model group.

As shown in Figure 3A, the MOD group achieved a significantly higher body weight than the CON group over the entire experimental period (*p* < 0.01). Compared with that in the MOD group, DHP administration resulted in a significant decrease in body weight (*p* < 0.01). To determine whether the DHP-induced weight decrease was due to reduced food intake, the food intake of mice in the MOD group and the DHP-L, DHP-M, and DHP-H groups was measured. As illustrated in Supplementary Figure S2A, the average daily food intake of mice in the MOD and DHP-L, DHP-M, and DHP-H groups was similar. The increased liver weight and liver index in MOD group were remarkably decreased (*p* < 0.05) following administration of DHP and PGZ (Figures 3B, C).

According to Figures 3D, E, the lobule structure of the liver lobules of mice in the CON group was clearly discernible, and the structure of the liver tissue was normal. The MOD group showed obvious fatty degeneration of hepatocyte tissue and excessive accumulation of lipid droplets and accompanied by the infiltration of inflammatory cells, suggesting that HFHF might cause severe liver injury in mice. Compared with MOD, DHP significantly reduced the fatty degeneration of hepatocytes, the infiltration of inflammatory cells, and local necrosis, which suggests that DHP ameliorated HFHF-induced NAFLD in the present mouse model.

### 3.3 Effect of *Dendrobium huoshanense* polysaccharide on the serum and liver lipid in the NAFLD mice

**FIGURE 4:**
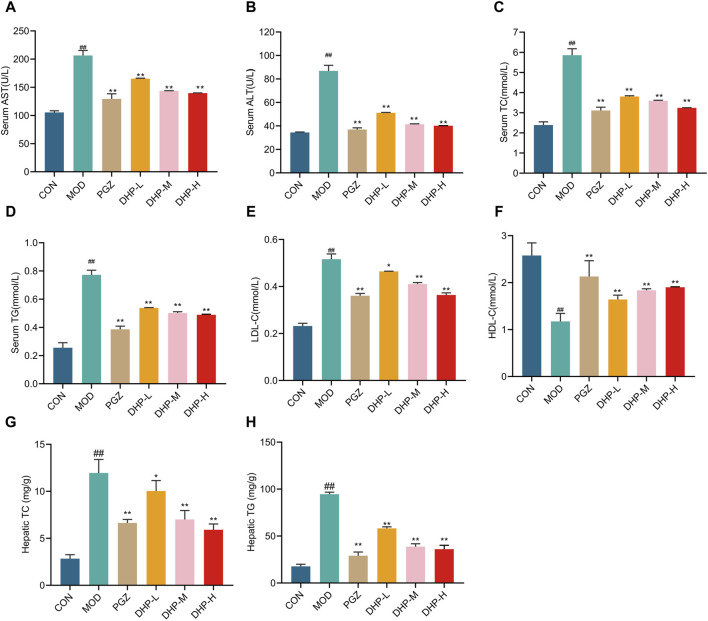
DHP improves biochemical indicator levels in HFHF-induced NAFLD mice. **(A)** Serum AST levels. **(B)** Serum ALT levels. **(C)** Serum total cholesterol (TC) levels. **(D)** Serum triglyceride (TG) levels. **(E)** Serum LDL-C levels. **(F)** Serum DHL-C levels. **(G)** Hepatic TC levels. **(H)** Hepatic TG levels. Data are presented as mean ± standard deviation (mean ± SD, n = 6). ^#^ *p* < 0.05, ^##^ *p* < 0.01 vs. the control group. *p < 0.05, **p < 0.01, vs. the model group.

Serum TG, TC, ALT, AST, LDL-C levels significantly increased while those of HDL-C levels obviously decreased in the MOD group compared with those in CON group (Figures 4A–F, *p* < 0.01). A similar trend was noticed for TG and TC levels in the liver, with the MOD group showing a higher level of these metabolites (Figures 4A–F, *p* < 0.01). Compared with those in the MOD group, the serum TG, TC, ALT, AST, and LDL-C levels were significantly decreased and those of HDL-C levels obviously increased following DHP or PGZ treatment; furthermore, the liver TG and TC levels were considerably reduced after DHP or PGZ treatment (Figures 4A–F, *p* < 0.01). These results indicate that DHP protects NAFLD mice from hepatic injury.

### 3.4 *Dendrobium huoshanense* polysaccharide reduced hepatic inflammation in NAFLD mice

**FIGURE 5:**
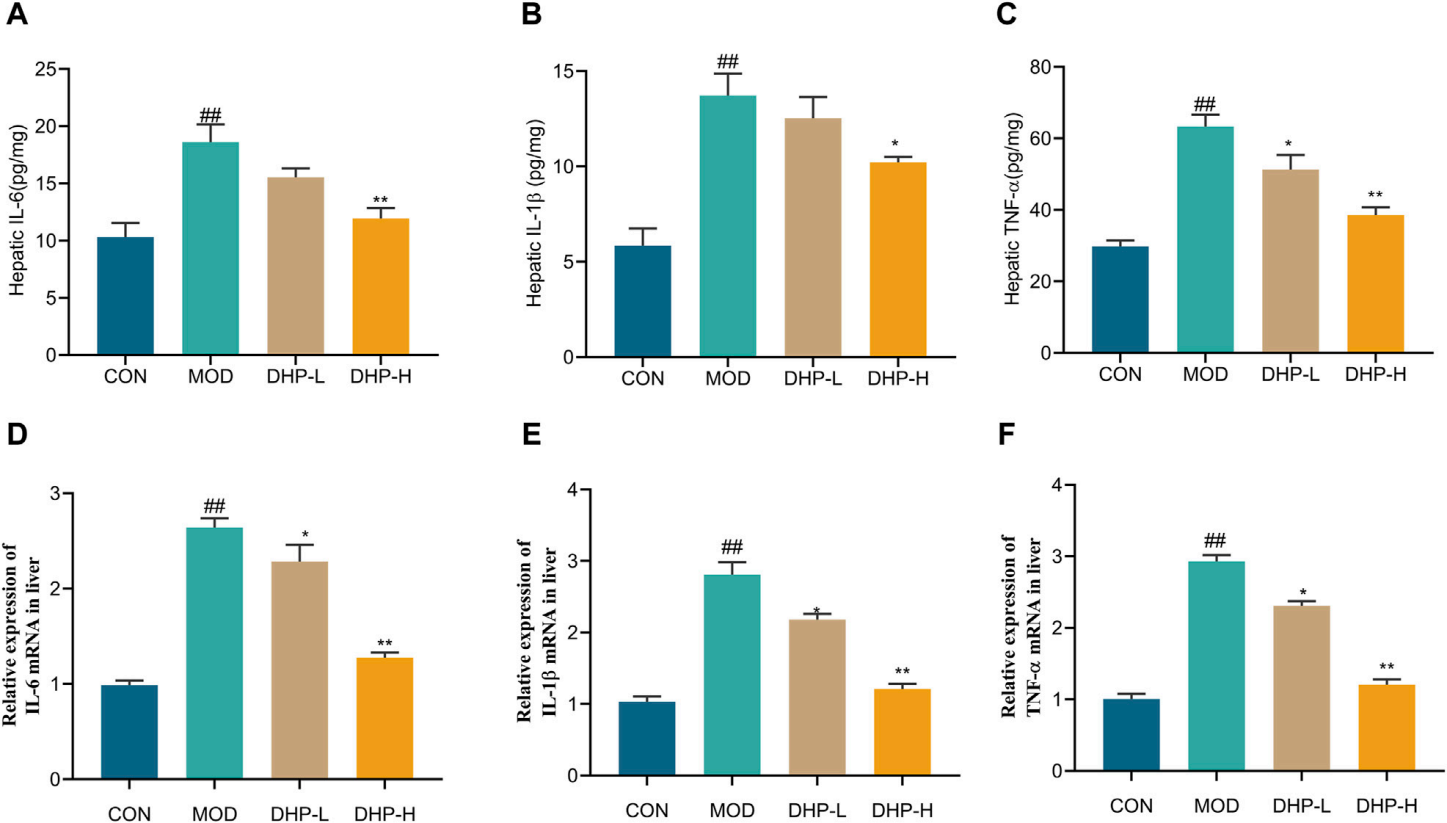
Anti-inflammatory properties of DHP on the HFHF-induced NAFLD. **(A–C)** The hepatic levels of IL-6, IL-1β, and TNF-α. **(D–F)** Liver mRNA expression levels of inflammatory response-related cytokines, including IL-6, IL-1β, and TNF-α, respectively. Data are presented as mean ± standard deviation (mean ± SD, n = 6). ^#^ *p* < 0.05, ^##^ *p* < 0.01 vs. the control group. *p < 0.05, **p < 0.01, vs. the model group.

As illustrated in Figures 5A–C, liver TNF-α, IL-6, and IL-1β levels in the mice fed HFHF remarkably increased (*p* < 0.01) compared with those in the CON group. Treatment with DHP-H effectively inhibited liver IL-1β, IL-6, and TNF-α expression compared with that in the MOD group. Compared with the CON group, the mRNA levels of TNF-α, IL-6, and IL-1β in the liver were elevated in the MOD group (*p* < 0.01). In comparison with the MOD group, DHP administration significantly reduced hepatic mRNA levels of IL-6, IL-1β, and TNF-α (*p* < 0.01) (Figure 5D–F).

**FIGURE 6:**
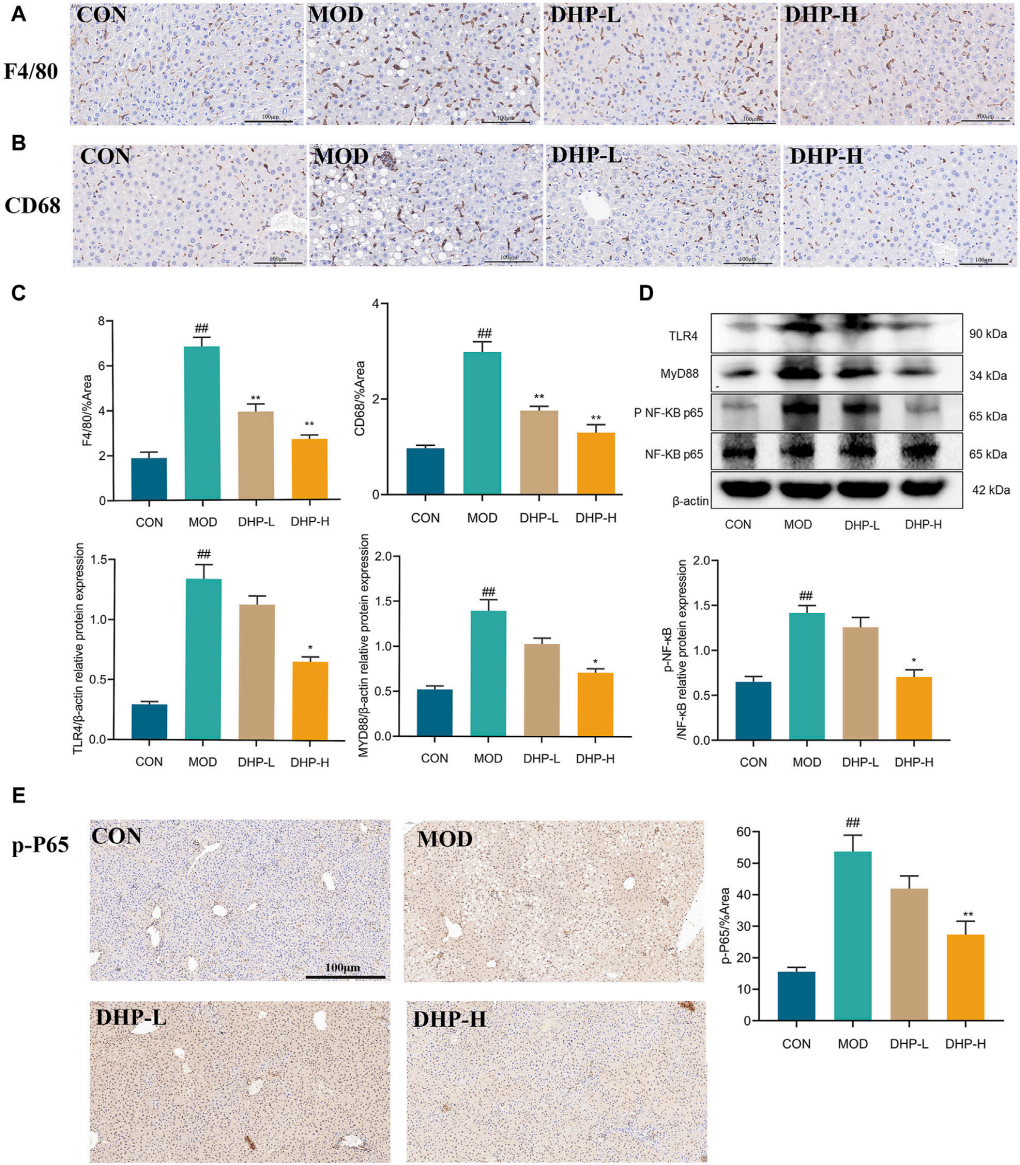
DHP alleviates liver inflammation in NAFLD mice. **(A–C)** Immunohistochemical staining of F4/80 and CD68 in liver tissue. **(D)** Representative immunoblot images of TLR4, MYD88, p-NF-κB protein in liver tissue. **(E)** Immunofluorescence staining of p-NF-κB in liver tissue. (scale 100 µm). Data are presented as mean ± standard deviation (mean ± SD, n = 3). ^#^ *p* < 0.05, ^##^ *p* < 0.01 vs. the control group. *p < 0.05, **p < 0.01, vs. the model group.

Immunohistochemical staining of F4/80 and CD68 showed that DHP reduced macrophage infiltration in the liver, further supporting the results of ELISA and PCR (Figure 6A, B). Furthermore, DHP treatment resulted in a significantly decreased in the expression of TLR4, Myd88, and p-NF-κB (Figure 6C). The results of the immunostructures were consistent with those of the Western blot (Figure 6D–E). These results show that DHP ameliorated HFHF diet-induced hepatic inflammation by modulating the TLR4/NF-κB signaling pathways.

### 3.5 Effect of *Dendrobium huoshanense* polysaccharide on liver metabolomic profiling

**FIGURE 7:**
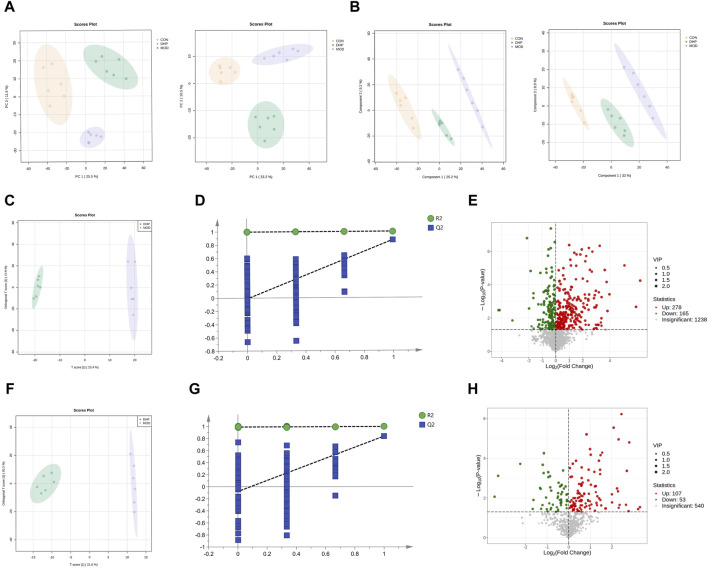
Liver metabolomic profiling by UPLC-QTOF/MS. **(A)** PCA score plot of the different groups in the positive ion (left) and negative ion (right). **(B)** PLS-DA score plot of the different groups in the positive ion (left) and negative ion (right). **(C)** Scores plots of OPLS-DA in the positive ion (MOD vs. DHP). **(D)** Permutation of OPLS-DA model in the positive ion (MOD vs. DHP). **(E)** The volcano plot in the positive ion (MOD vs. DHP). **(F)** Scores plots of OPLS-DA in the negative ion (MOD vs. DHP). **(G)** Permutation of OPLS-DA model in the negative ion (MOD vs. DHP). **(H)** The volcano plot in the negative ion (MOD vs. DHP). Data are presented as mean ± standard deviation (mean ± SD, n = 6).

A further metabolomics study was conducted in order to determine the effect of DHP on liver metabolic responses in NAFLD mice. The liver metabolic profiles were obtained using UHPLC-MS/MS. In the metabolomics study, 4,146 peaks were identified (the counts of ESI + ions were 2,859 and 1,287, respectively). The PCA and PLS-DA results indicated distinguishable liver metabolites among the CON, MOD, and DHP groups (Figures 7A, B). According to the PCA and PLS-DA score plots, good aggregation was observed among the CON, MOD, and DHP groups, indicating good reliability of the method. Furthermore, as shown in Figure 7C, F, supervised OPLS-DA was used to compare the MOD and DHP groups in order to better distinguish between these groups and improve the effectiveness and analytical capability of the model. By examining the OPLS-DA score plot, it was demonstrated that there was a significant difference between the MOD and DHP groups in the positive/negative model, indicating that the metabolites were different. These results indicate that DHP can regulate metabolic disorders caused by feeding HFHF in the present mouse model.

Based on the positive/negative ion mode, the R2 and Q2 results were (0.997, −0.013) (Figure 7D) and (0.991, −0.079) (Figure 7G). The results showed that the model was reliable and had predictive ability. Based on the volcano plot, we found that compared with the model group, 165 metabolites were dramatically decreased and 278 metabolites were significantly increased in the positive ion mode after DHP treatment; and 53 metabolites were greatly decreased and 107 metabolites were significantly increased in negative ion mode following DHP treatment (Figures 7E–H).

### 3.6 Changes in metabolic pathways in fed HFHF-fed mice induced by D*endrobium huoshanense* polysaccharide

**FIGURE 8:**
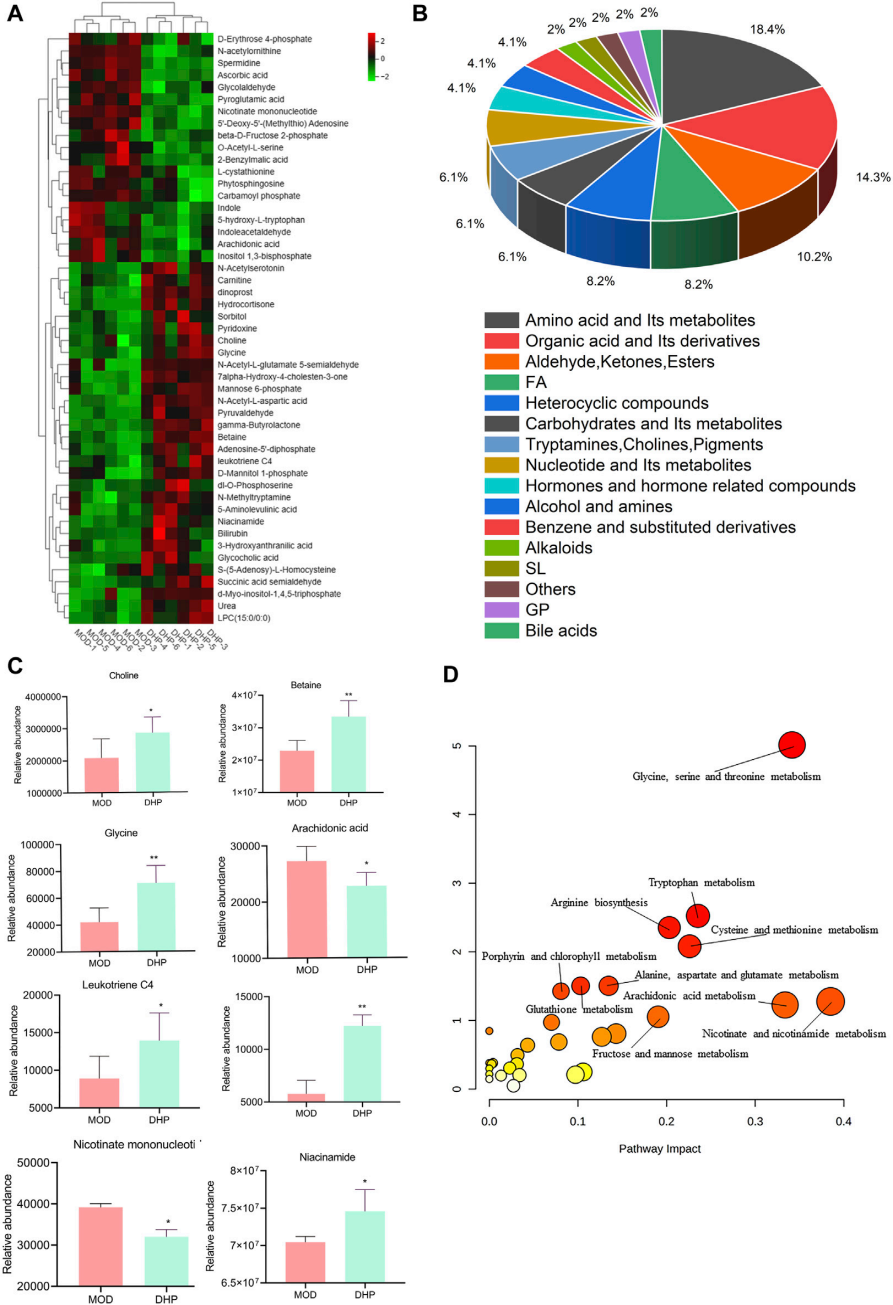
Metabolic alterations between the MOD groups and DHP groups. **(A)** Heatmap of different metabolites. **(B)** The percentages of major metabolites are illustrated in a pie chart. **(C)** Eight representative differentially expressed metabolites **(D)** KEGG pathway analysis of metabolic pathway enrichment about differentially abundant metabolites.

Using untargeted metabolomics, 49 differential liver in the liver were determined between the MOD groups and the DHP groups. These metabolites were identified by screening with the following screening criteria: *p* < 0.05, VIP >1, and KEGG database searches (Table 2). Compared with the MOD group, 19 metabolites were obviously decreased and 30 metabolites were significantly increased in the DHP group (Figure 8A). The main categories for these 49 differential metabolites were as follows: amino acids and its metabolites (18.36%); organic acids and its derivatives (14.28%); aldehydes, ketones, esters (10.20%); and fatty acids (8.16%) (Figure 8B). MetabolAnalyst 5.0 was used to performed KEGG-based metabolic pathway enrichment analyses of various liver metabolites to identify the major metabolic pathways affected in the MOD and DHP groups. Results showed that these different metabolites primarily enriched in glycine, serine, and threonine, nicotinate and nicotinamide metabolism, and arachidonic acid metabolism, among others (Figures 8C, D).

**TABLE 2:** Differential metabolites in NAFLD model mice after the treatment of DHP.

Name	Formula	m/z	RT (min)	DHP VS MOD *p*-value	Type
Niacinamide	C_6_H_6_N_2_O	122.05	1.41	0.02	up
Pyroglutamic acid	C_5_H_7_NO_3_	129.04	0.78	9.92E-04	down
S-(5-Adenosy)-L-Homocysteine	C_14_H_20_N_6_O_5_S	384.12	1.23	0.04	up
d-Myo-inositol-1,4,5-triphosphate	C_6_H_15_O_15_P_3_	419.96	12.33	0.01	up
leukotriene C4	C_30_H_47_N_3_O_9_S	625.30	2.17	0.03	up
Succinic acid Semialdehyde	C_4_H_6_O_3_	102.03	1.57	0.01	up
N-acetylornithine	C_7_H_14_N_2_O_3_	174.10	5.72	1.07E-03	down
Choline	C_5_H_14_NO+	104.11	0.76	0.04	up
N-Methyltryptamine	C_11_H_14_N_2_	174.12	4.74	0.01	up
D-Erythrose 4-phosphate	C_4_H_9_O_7_P	200.01	0.70	0.05	down
Urea	CH_4_N_2_O	60.03	0.83	8.00E-04	up
L-cystathionine	C_7_H_14_N_2_O_4_S	222.07	0.84	0.02	down
Glycine	C_2_H_5_NO_2_	75.03	0.75	1.96E-03	up
Phytosphingosine	C_18_H_39_NO_3_	317.29	5.90	0.02	down
Dinoprost	C_20_H_34_O_5_	354.24	7.34	3.20E-06	up
3-Hydroxyanthranilic acid	C_7_H_7_NO_3_	153.04	5.18	0.04	up
O-Acetyl-L-serine	C_5_H_9_NO_4_	147.05	1.13	0.01	down
Arachidonic acid	C_20_H_32_O_2_	304.24	12.11	0.01	down
Hydrocortisone	C_21_H_30_O_5_	362.21	5.52	6.53E-04	up
Glycocholic acid	C_26_H_43_NO_6_	465.31	6.07	0.04	up
Carnitine	C_7_H_15_NO_3_	161.11	0.84	0.01	up
7alpha-Hydroxy-4-Cholesten-3-one	C_27_H_44_O_2_	400.33	7.26	3.54E-04	up
Nicotinate mononucleotide	C_11_H_15_NO_9_P+	336.05	5.72	3.27E-05	down
5′-Deoxy-5'-(Methylthio) Adenosine	C_11_H_15_N_5_O_3_S	297.09	2.81	3.29E-04	down
Glycolaldehyde	C_2_H_4_O_2_	60.02	12.16	3.40E-03	down
Spermidine	C_7_H_19_N_3_	145.16	12.40	1.83E-06	down
N-Acetylserotonin	C_12_H_14_N_2_O_2_	218.11	3.12	0.01	up
Indole	C_8_H_7_N	117.06	2.38	0.03	down
5-Aminolevulinic acid	C_5_H_9_NO_3_	131.06	1.41	0.01	up
Inositol 1,3-bisphosphate	C_6_H_14_O_12_P_2_	340.00	12.23	3.19E-03	down
Betaine	C_5_H_11_NO_2_	117.08	0.87	2.11E-03	up
gamma-Butyrolactone	C_4_H_6_O_2_	86.04	1.82	9.26E-04	up
LPC(15:0/0:0)	C_23_H_48_NO_7_P	481.32	8.28	1.99E-03	up
N-Acetyl-L-aspartic acid	C_6_H_9_NO_5_	175.05	1.16	2.86E-06	up
Pyruvaldehyde	C_3_H_4_O_2_	72.02	1.15	4.29E-04	up
Pyridoxine	C_8_H_11_NO_3_	169.08	1.87	0.04	up
Bilirubin	C_33_H_36_N_4_O_6_	584.26	7.75	0.06	up
Carbamoyl phosphate	CH_4_NO_5_P	140.98	2.46	0.01	down
Adenosine-5′-diphosphate	C_10_H_15_N_5_O_10_P_2_	427.03	1.15	2.15E-03	up
Ascorbic acid	C_6_H_8_O_6_	176.03	1.13	2.12E-04	down
Sorbitol	C_6_H_14_O_6_	182.08	1.29	0.02	up
5-hydroxy-L-tryptophan	C_11_H_12_N_2_O_3_	220.09	2.38	0.02	down
Indoleacetaldehyde	C_10_H_9_NO	159.07	2.39	2.18E-03	down
Dl-O-Phosphoserine	C_3_H_8_NO_6_P	185.01	4.00	0.05	up
D-Mannitol 1-phosphate	C_6_H_15_O_9_P	262.05	1.84	0.04	up
N-Acetyl-L-glutamate 5-Semialdehyde	C_7_H_11_NO_4_	173.07	1.17	0.02	up
Beta-D-Fructose 2-Phosphate	C_6_H_13_O_9_P	260.03	0.86	0.02	down
Mannose 6-phosphate	C_6_H_13_O_9_P	260.03	1.06	2.18E-03	up
2-Benzylmalic acid	C_11_H_12_O_5_	224.07	3.52	0.01	down

## 4 Discussion

The rate of incidence of NAFLD, already one of the most prevalent liver diseases in the world, is increasing. Traditional Chinese medicine is considered to exert beneficial against NAFLD through various approaches, including regulation of lipid metabolism disorders, improvement in intestinal flora, and reduction of insulin resistance and inflammation (Zhang et al., 2022; Li Q. et al., 2023; Ding et al., 2023). Our results show that DHP has a characteristic polysaccharide peak with a molecular weight of 179.935 kDa and its main components are Man and Glc with a molar ratio of 68.97:31.03. The normalized liver function markers and decreased liver lipid levels demonstrated that DHP, as expected, exerted a substantial hepatoprotective effect against NAFLD. Our study revealed that DHP intervention effectively reduced the HFHF-induced body weight gain, liver weight, and liver index in mice. Furthermore, DHP treatment was able to normalized serum TG, TC, ALT, AST, LDL-C, HDL-C, as well as liver TC and TG levels following treatment with DHP(Figure 4). Moreover, H&E staining and oil red O staining showed that DHP-treated mice exhibited obvious reduction in lipid droplets and inflammation (Figures 3D, E). Through integrated analysis of ELISA, western blot, immunohistochemical staining, and metabolomics, we confirmed that the hepatoprotective effect of DHP was largely based on regulation the TLR4-NF-κB pathway; amelioration of the liver inflammatory response; and metabolism of glycine, serine, and threonine, nicotinate and nicotinamide, and arachidonic acid (Kundi et al., 2021). Our results are consistent with those of previous studies, that is, DHP treatment relieved abnormal and disordered lipid metabolism (Li F. et al., 2023). According to these findings, DHP significantly ameliorated liver injury after it was administered to mice with HFHF-induced NAFLD, thereby reversing the harmful effects of HFHF.

Inflammation plays a fundamental role in the progression of NAFLD. Accumulation of liver lipids and activation of macrophages as well as the accumulation of liver lipids influences the release of the proinflammatory factors leukotriene-6, tumor necrosis factor-alpha, and interleukin-1, which result in liver inflammation, steatosis, and cell injury (Afonso et al., 2021; Ta et al., 2023). In addition, researchers have shown that the pathological processes involved in NAFLD can be alleviated by inhibiting macrophage infiltration and its release of inflammatory factors (Henao-Mejia et al., 2012; Yang et al., 2015). In particular, high levels of TNF-α not only injure hepatocytes but also stimulate the production of other inflammatory cytokines and lead to hepatocyte apoptosis (Tian et al., 2021). We found that DHP significantly reduced the levels of pro-inflammatory cytokines (TNF-α, IL-6, and IL-1β) and macrophage cell infiltration in the liver. These findings indicate that DHP may enhance anti-inflammatory responses (Figure 5; Figures 6A, B).

According to some reports, the TLR4-/NF-κB pathway is responsible for the inflammation of the liver associated with NAFLD (Henao-Mejia et al., 2012). A major component of the TLR family of cell surface pattern recognition receptors, TLR4 plays a vital role in the immune response of the body as well as the inflammatory response of the body since it is one of the essential components of the TLR family (Henao-Mejia et al., 2012). When hepatocytes are stimulated, TLR4 activates downstream MyD88, which ultimately leads to NF-κB activation and nuclear translocation, inducing the release of inflammatory cytokines and cellular damage (Kuzmich et al., 2017; Bruneau et al., 2021). To further explore the potential mechanism underlying the effect of DHP on the inflammatory response in NAFLD, we evaluated the TLR4/NF-κB pathway. In this study, DHP significantly inhibited the aberrant expression of TLR4/NF-κB pathway proteins, suggesting that DHP may attenuate the inflammatory response in NAFLD through this pathways (Figures 6C–E).

Analysis of the differences in metabolites between the DHP and MOD groups was performed using UPLC-QTOF/MS in order to investigate the mechanism of action of DHP against NAFLD. The metabolic pathways of different metabolites were enriched and analyzed to identify significant metabolic pathways associated with NAFLD. The results of this experiment indicated that DHP may alleviate NAFLD through glycine, serine, and threonine, nicotinate and nicotinamide metabolism, and arachidonic acid metabolism (Figure 8D).

Recent metabolomics-based studies have found that disturbances in amino acid metabolism play a crucial role in the pathogenesis of NAFLD, with a reduction in glycine concentrations found to be negatively associated with hepatocellular dilatation and hepatic lobular inflammation (Yamakado et al., 2017; Gaggini et al., 2018). Choline is a critical nutrient that plays a vital role in human health and is required for the synthesis of betaine, acetylcholine and phospholipids (Leermakers et al., 2015). There is increasing experimental evidence that choline and betaine reduce inflammation and steatosis in the liver. (Wang et al., 2013; Aron-Wisnewsky et al., 2020; Cheng et al., 2021). In this study, glycine, choline, and betaine were significantly elevated in the DHP group compared with that in the MOD group (Figure 8C); these findings were consistent with previously reported results for glycine, serine, and threonine in NAFLD (Rom et al., 2020).

Nicotinic acid is converted into nicotinamide by transamination, which is one of the primary precursors of the naturally occurring nicotinamide adenine dinucleotide (NAD+)(Zhai et al., 2009). During NAD + synthesis, niacinamide (NAM) is catalyzed by phosphoribosyltransferase (NAMPT) to generate nicotinamide mononucleotide (NMN) which is converted to NAD + by the mononucleotide transferase nicotinamide adenosine (Sampath et al., 2015). Nicotinamide or related metabolites have been reported to ameliorate the inflammatory response in NAFLD (Zhuang et al., 2017). The present study suggests that the therapeutic effects of DHP against NAFLD involved an increase in the levels of nicotinamide, enhanced NAD + biosynthesis, and regulation of nicotinic acid and nicotinamide metabolism.

**FIGURE 9:**
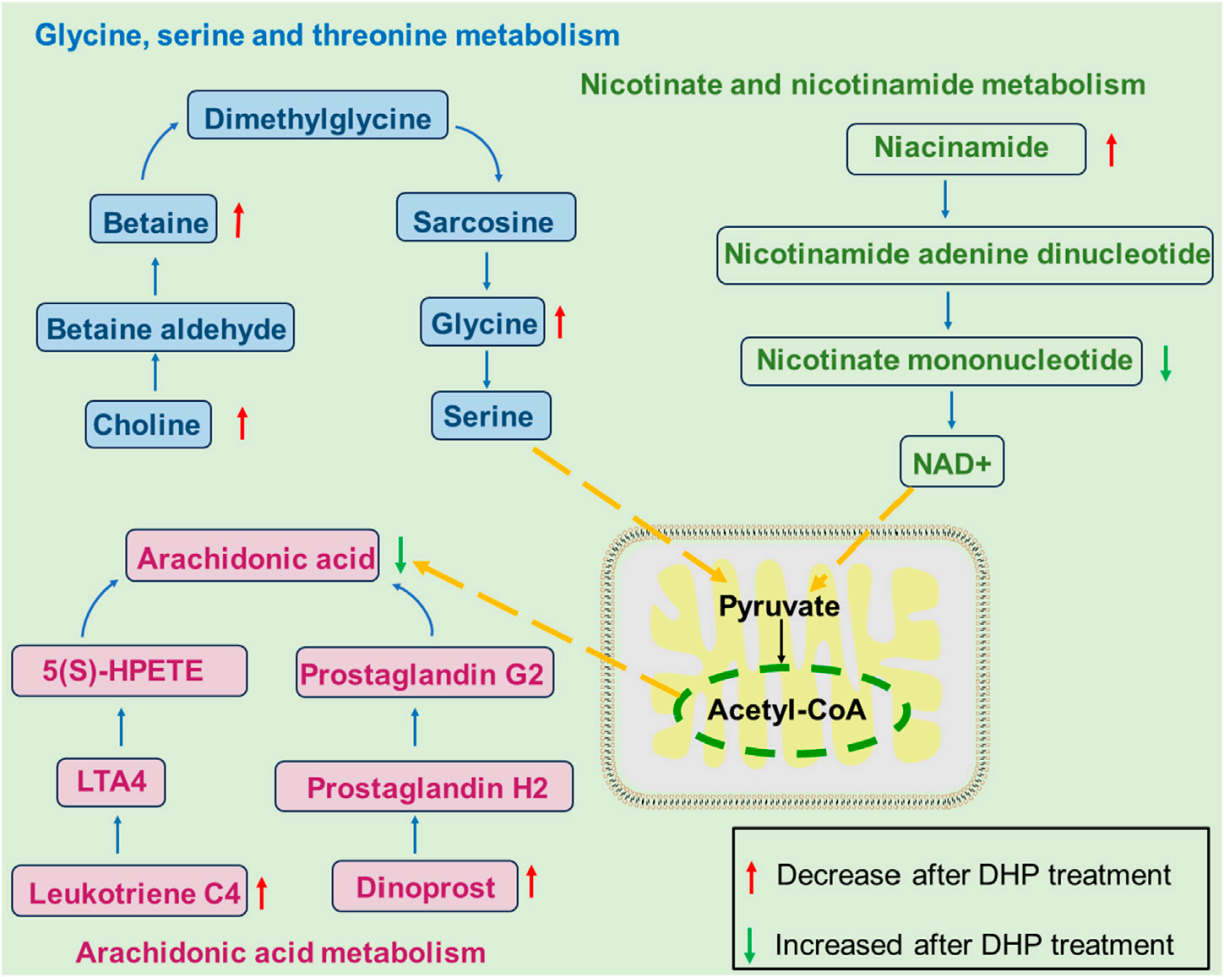
Changes in metabolic pathways in NAFLD model mice treated with DHP. The therapeutic effects of DHP against NAFLD may involve modulation of betaine, glycine, and choline in the glycine, serine, and threonine metabolic pathways; NAM and NMN in nicotinic acid and nicotinamide metabolism, as well as arachidonic acid, leukotriene C4, and dinoprost in arachidonic acid metabolism.

Furthermore, in the arachidonic acid metabolic pathway, arachidonic acid accelerates the progression of hepatotoxicity and increases eicosanoid levels, which are correlated with the release of pro-inflammatory cytokines and reactive oxygen species being released during NAFLD-related inflammation (Sen et al., 2020). Arachidonic acid levels were found to be significantly increased in the MOD group, which suggests that impaired arachidonic acid metabolism is associated with NAFLD. The therapeutic effects of DHP against NAFLD may involve modulation of betaine, glycine, and choline in the glycine, serine, and threonine metabolic pathways; NAM and NMN in nicotinic acid and nicotinamide metabolism, as well as arachidonic acid, leukotriene C4, and dinoprost in arachidonic acid metabolism, as shown in Figure 9.

Among these metabolites, glycine has been reported to reduce liver inflammation and ameliorate NAFLD by downregulating the TLR4 signaling pathway (Yang et al., 2017; Qu et al., 2023). There is increasing experimental evidence that choline and betaine reduce inflammation and steatosis in the liver, which may be correlated with their regulation of the TLR4 pathway (Wang et al., 2013; Aron-Wisnewsky et al., 2020; Cheng et al., 2021). Nicotinamide or related metabolites have been reported to ameliorate the inflammatory response in NAFLD by regulating the TLR4 pathway (Zhuang et al., 2017).

In this study, we found that DHP significantly reversed the abnormal levels of the HFHF diet-induced metabolites choline, glycine, betaine, NAM, MNN, and arachidonic acid, thereby interfering with the metabolism of glycine, serine, threonine; nicotinate and nicotinamide metabolism; and arachidonic acid metabolism, as well as modulating TLR4-/NF-κB pathway, thereby effectively treating NAFLD. Despite these contributions, the present study has some shortcomings and requires further validation and analysis of differential metabolites. Additional *in vivo* and *in vitro* studies of the identified metabolites and pathways and related biological processes are necessary.

## 5 Conclusion

In summary, this study indicates that DHP exerts a protective effect against NAFLD. Using an untargeted metabolomics approach, we found that the action of DHP on NAFLD may involve regulation of the metabolism of glycine, serine, threonine, nicotinate and nicotinamide, as well as arachidonic acid metabolism and inhibition of the TLR4 signaling pathway. This study provides a theoretical basis for the therapeutic utilization of DHP in the prevention and control of NAFLD, with promising clinical applications.

## Data Availability

The raw data contained in the article can be found at: https://www.jianguoyun.com/p/DYvhY_QQm-fRDBjEocYFIAA.
