# Supplementary figures and images for: Untargeted metabonomics and TLR4/ NF-κB signaling pathway analysis reveals potential mechanism of action of Dendrobium huoshanense polysaccharide in nonalcoholic fatty liver disease
Source: Front Pharmacol. 2024 Jun 3;15:1374158. doi: 10.3389/fphar.2024.1374158 (PMC11180771; doi:10.3389/fphar.2024.1374158)

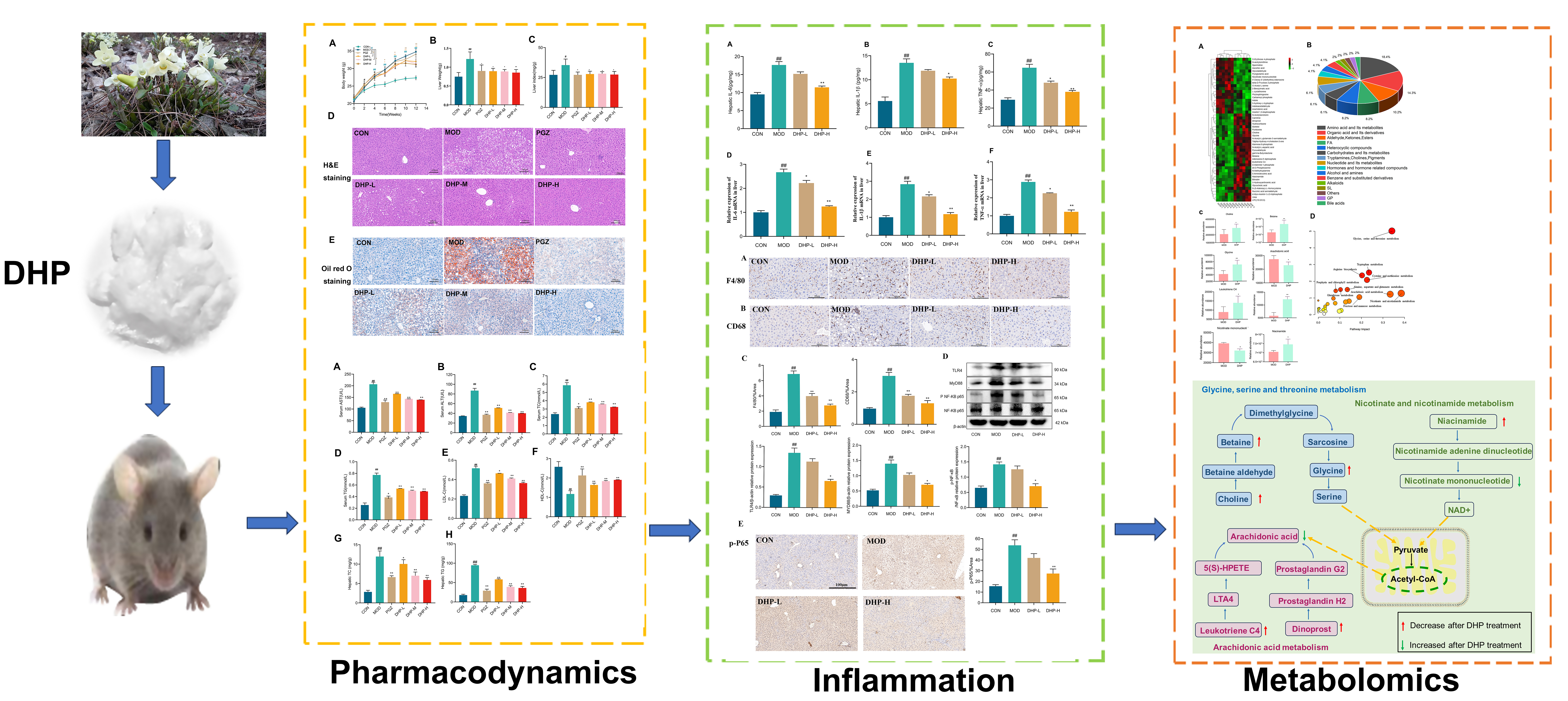

Supplement: Supplementary file 1 [file Image1.jpg]
