# Supplementary material for: Untargeted metabonomics and TLR4/ NF-κB signaling pathway analysis reveals potential mechanism of action of Dendrobium huoshanense polysaccharide in nonalcoholic fatty liver disease
Source: Front Pharmacol. 2024 Jun 3;15:1374158. doi: 10.3389/fphar.2024.1374158 (PMC11180771; doi:10.3389/fphar.2024.1374158)
Supplement: Supplementary file 2 [file DataSheet1.docx]

Figure S1. The details are as follows: The surface morphological features of DHP were scanned using a scanning electron microscope (Hitachi Regulus, JAPAN). The DHP was mixed with KBr, ground, and then subjected to FT-IR measurements in the wavelength range of 400-4000 cm^-1^ (Thermo Electron, USA). The homogeneity and molecular weight of various fractions were measured using SEC-MALLS-RI. The weight and number-average molecular weight (Mw and Mn) and polydispersity index (Mw/Mn) of various fractions in 0.1 M NaNO_3_ aqueous solution containing 0.02% NaN_3_(or DMSO solution containing 0.5% LiBr)were measured on a DAWN HELEOS-II laser photometer Wyatt Technology Co., USA) equipped with two tandem columns (300 × 8 mm, Shodex OH-pak SB-805 and 803; Showa Denko K.K., Tokyo, Japan) (or three tandem columns (300 × 8 mm, Shodex OH-pak SB-805, 804 and 803; Showa Denko K.K., Tokyo, Japan)) which was held at 45℃ (or 60℃) using a model column heater by Sanshu Biotech. Co., LTD (Shanghai，China). The flow rate is 0.6 mL/min (or 0.3 mL/min). A differential refractive index detector (Optilab T-rEX, Wyatt Technology Co., USA) was simultaneously connected to give the concentration of fractions and the dn/dc value. The dn/dc value of the fractions in 0.1 M NaNO_3_ aqueous solution containing 0.02% NaN_3_ was determined to be 0.141 mL/g, and in DMSO solution was determined to be 0.07 mL/g. Data were acquired and processed using ASTRA6.1 (Wyatt Technology). The monosaccharide composition of DHP was determined by ion chromatography (IC). In an ampoule, 5 mg of DHP was hydrolyzed with 2 M TFA at 121℃ for 2 h, and then eluted with ultrapure water and 100 mM NaOH at a flow rate of 0.5 mL/min. Standard monosaccharides were determined in the same way.


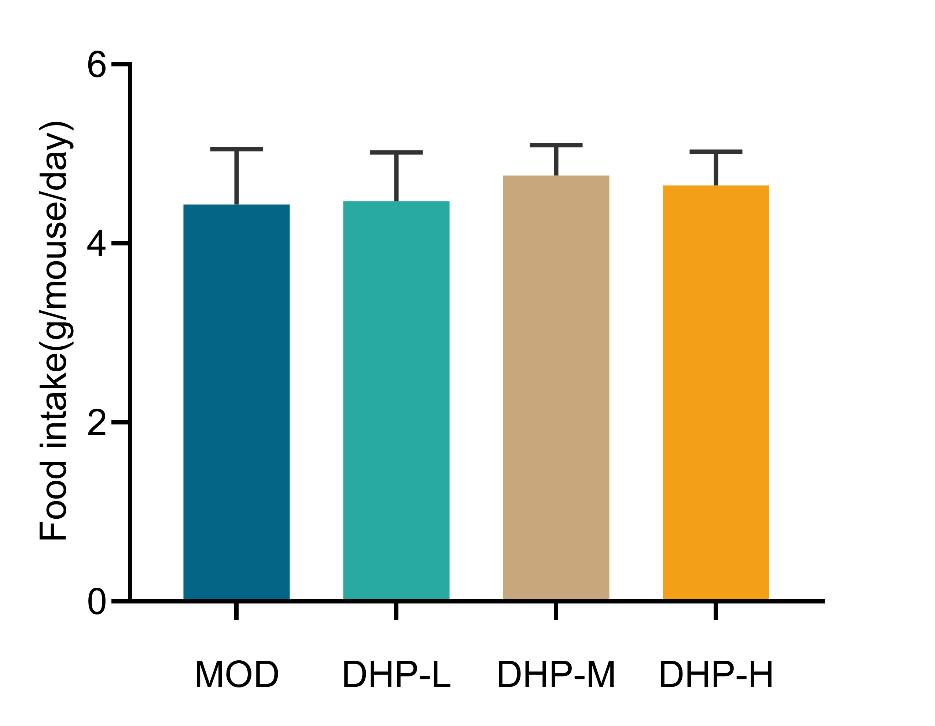


Figure S2A The average food intake amount of MOD and DHP-L, DHP-M, and DHP-H groups mice during the experimental feeding period of 12 weeks.


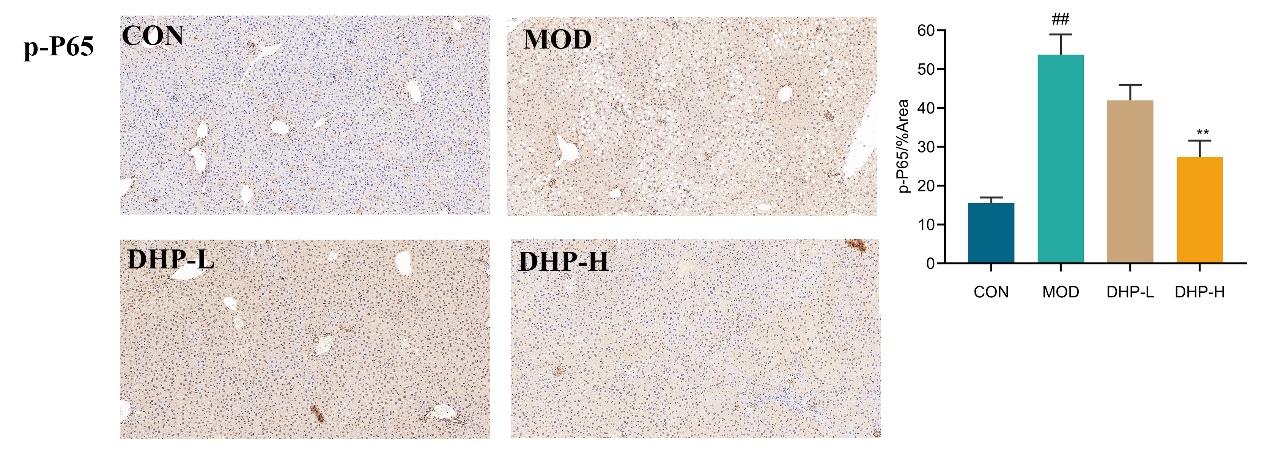
 Figure S3A Immunohistochemical staining of p-NF-κB in liver tissue.
